# Phytoconstituents of *Chloranthus elatior* as a potential adjunct in the treatment of anxiety disorders: In vivo and in silico approaches

**DOI:** 10.1016/j.heliyon.2024.e40728

**Published:** 2024-11-28

**Authors:** Umme Tabassum Arobi Katha, Yesmin Begum, Md Golam Mortuza, Sayma Sharmin, Md Rafiquzzaman, Suvro Biswas, Md Abu Saleh

**Affiliations:** a Department of Pharmacy, Southeast University, Dhaka, Bangladesh; b Department of Science and Humanities, Bangladesh Army International University of Science and Technology, Cumilla, 3500, Bangladesh; c Department of Pharmacy, Jahangirnagar University, Savar, Dhaka, Bangladesh; d Microbiology Laboratory, Department of Genetic Engineering and Biotechnology, University of Rajshahi, Rajshahi-6205, Bangladesh

**Keywords:** *Chloranthus elatior*, Phytochemical, Anxiolytic, Sedative, Docking and ADMET

## Abstract

Traditional plants have played a vital role in civilization and medicine throughout history. *Chloranthus elatior*, a plant used in South Asian traditional medicine, has various medicinal applications but limited research on its impact on the central nervous system (CNS). This study analyzed the methanolic leaf extract of *Chloranthus elatior* (MECE) for secondary metabolites and conducted experiments to evaluate the sedative, and anxiolytic effect of MECE on a mice model. To assess anxiolytic effects, elevated plus maze (EPM) and light-dark box (LDB) tests were performed. Sedative effects were explored in open field and hole-cross tests. Additionally, *in silico* investigations included molecular docking and ADME/T property assessments for 40 secondary metabolites. The phytochemical analysis of MECE revealed the presence of alkaloids, tannins, flavonoids, and glycosides. MECE exhibited significant anxiolytic effects in both the EPM and LDB tests, with statistical significance (P < 0.001). The open field and hole-cross tests demonstrated significant sedative potential (P < 0.05) compared to the standard Diazepam. Furthermore, molecular docking was performed to evaluate the potential of the compounds with a potassium channel protein. Among them, Chloramultilide C, 4-dimethoxyflavanone, and Neolitacumone B were identified as potential against the target protein with a binding score of −8.8 kcal/mol, −6.5 kcal/mol, and −6.4 kcal/mol, respectively. Additionally, pharmacokinetic attributes and ADMET analysis emphasized promising properties for drug development. These findings suggest that MECE possesses sedative and anxiolytic properties that could be valuable for addressing insomnia and anxiety associated with various psychiatric disorders.

## 1 Introduction

Anxiety is a central nervous system (CNS) disorder characterized by excessive worry, difficulty concentrating, and physiological symptoms such as muscle contractions, insomnia, and cognitive impairment. It can have significant social, economic, and personal implications. Moreover, the morbidity rate connected with anxiety is exceptionally high [1]. According to extensive community-based surveys, anxiety disorders affect up to 33.7 percent of the population at some point in their lives. There is evidence that certain anxiety disorders are underdiagnosed and undertreated, with little change in prevalence rates in recent years [2,3]. During the COVID-19 pandemic, anxiety and sleep disturbances were reported in 26.9 % and 27.6 % of people worldwide, respectively [4]. Treatments for anxiety and insomnia, such as benzodiazepines, barbiturates, meprobamate, and buspirone, often come with numerous negative side effects, such as amnesia, muscle relaxation, dependency, and tolerance [5].

In contrast, plants have been shown to be a plethora reservoir of novel chemicals with pharmacological properties, gaining importance in the search for more effective medicines [6]. Approximately 25 % of existing medicines are derived from plants, with many more being synthetic derivatives based on plant compounds [7]. Psychological disorders often co-occur with other mental illnesses, increasing the burden and healthcare cost. Mood, anxiety, and sleep disorders have historically been treated with herbal remedies [8,9]. Additionally, plant products serve as valuable raw materials for creating a wide range of conventional and modern drugs, offering developing countries alternative sources of revenue, employment, and foreign currency. Phyto-medicine often serves as the primary or only affordable healthcare system in many impoverished nations. As a complement or replacement of existing anxiolytics, plant-based "phytomedicines" could offer innovative therapeutic approaches. In this context, preliminary phytochemical screening and anxiolytic evaluation of the methanolic extract of *C. elatior*, a member of the Chloranthceae family, were conducted. *C. elatior,* a perennial shrub, has limited research on its leaf extract. Moreover, the Chloranthaceae family is known for its diverse array of bioactive chemicals, particularly sesquiterpenoids, which possess significant activities such as sedative, anti-inflammatory, anti-tumor effects, and more [10,11].

The previous phytochemical analysis investigation discovered the presence of alkaloids, flavonoids, terpenoids, saponins, quinones, glycosides, and steroids in this plant. One study demonstrates that *C. elatior* solvent extract includes therapeutically relevant bioactive components, bolstering the usage of plant species as traditional medicine for the treatment of a variety of ailments [12]. Folklore medicine has historically depended on plants with medicinal properties to treat mental and neurological ailments. It is a popular folklore medication among indigenous tribes. Another researcher has documented the traditional use of this plant in curing localized pain, swelling, skin inflammation, wound healing, joint pain, body aches, and fever [13]. In previous investigations, researchers focused on the antipyretic, anti-inflammatory, analgesic, and antifungal activity of *C. elatior* plants. However, no complete sedative or anxiolytic assessments or scientific reports on *C. elatior* have been published so far [[14], [15], [16]].

The field of drug discovery and development has rapidly advanced, incorporating various tools and techniques, including molecular dynamics simulations, molecular docking, molecular modeling, and machine learning. These advancements enable the identification of potential therapeutic targets for various diseases, reducing both the time and costs associated with drug development [[17], [18], [19]]. This research primarily focused on the sedative and anxiolytic effects of *C. elatior* as well as virtual analysis through molecular docking based on pharmacological properties to predict the mechanisms underlying the significant pharmacological properties of the selected extract and identify specific binding sites related to pharmacological actions.

## 2 Materials and methods

### 2.1 Plant collection

After conducting a comprehensive literature review, we selected *C. elatior*, a plant belonging to the Chloranthaceae family, as the subject of our study. Plant specimens of *C. elatior* were collected in March 2021 from the Botanical Garden in Dhaka, Bangladesh. A professional taxonomist from the National Herbarium of Bangladesh identified the plant after its collection. The *C. elatior* specimen has been assigned the accession number 64985.

### 2.2 Plant extract preparation

The plant material underwent a drying process in the shade until all the moisture had evaporated. Subsequently, approximately 250 g of coarse leaf powder was macerated in a 96 percent aqueous-methanol solution at room temperature for a duration of 17 days. This maceration took place in a container with its contents sealed using aluminum foil, with periodic gentle shaking. The extract was filtered using fresh cotton plugs and Whatman No. 1 filter paper. The filtrate extract was then concentrated using a rotary evaporator under reduced pressure at a temperature of 55 °C to obtain a crude extract. Following this step, the filtrate was subjected to a steam bath to transform it into a semi-solid or thick paste, which was subsequently dried at a low temperature of 64 °C. The resulting thick paste of *C. elatior* crude extract was completely solubilized in a normal saline solution (0.9 percent w/v).

### 2.3 Phytochemical screening

To identify the presence of alkaloids, flavonoids, tannins, saponins, steroids, cardiac glycosides, and carbohydrates, the methanolic extract of *C. elatiors* leaves was qualitatively assessed using standard techniques. The presence of alkaloids in the methanolic extract of *C. elatiors* leaves was determined using ‘Wagner's test’ [20], ‘Dragendorff's test’ [21], and ‘Mayer's test’ [21]. A brown-colored precipitate, an orange-red precipitate, and a yellowish or white precipitate indicated the prese

nce of alkaloids, respectively. Additionally, the existence of flavonoids was tested using ‘Shinoda's test’ [22], ‘Sodium hydroxide test’ [23], and ‘Pew's test’ [24]. A deep yellow color followed by colorless after adding a few drops of dilute HCL, and red color, respectively, showed the presence of flavonoids. Furthermore, the presence of tannins was confirmed by the ‘Ferric chloride test’ [25] and ‘Lead sub-acetate test’ [25]. A grey/black color formation and a cream gelatinous precipitate displayed positive results, respectively. Besides, saponins were also identified in the extracted sample using the ‘Foam test’ [26], where the formation of stable foam presented positive results. Moreover, the steroids test was conducted using the ‘Salkowski test’ [27], and the cardiac glycosides and carbohydrate test were performed using the ‘Keller-Kiliani test’ [28] and ‘Molisch's test’ [25], respectively. A brown ring formation confirmed the existence of cardiac glycosides, while a purple-colored product formation confirmed the existence of carbohydrates.

### 2.4 Experimental animals

Swiss albino male mice weighing between 20 and 25 g were procured from Jahangirnagar University in Savar, Dhaka, Bangladesh. These animals were housed under standard conditions, including a 12-h light/dark cycle (from 7 a.m. to 7 p.m.), an ambient temperature of 25 °C, and a relative humidity of 55–65 percent. Flaking wood pellets were used as bedding, and their regular diet consisted of a standard food designed by Jahangirnagar University, along with access to clean water ad libitum. A 14-day acclimatization period was observed before conducting any experiments involving the animals. All experimental procedures involving animals adhered to the guidelines provided in the National Institutes of Health's Guide for the Care and Use of Laboratory Animals (1978). The animal testing protocol was approved by the Animal Use and Care Committee of Southeast University. Cages were cleaned and disinfected three times a week, with each standard cage housing five mice.

### 2.5 Experimental design

It is widely recognized that benzodiazepines exhibit anxiolytic properties at low dosages, while higher doses induce drowsiness and muscle relaxation. As a standard control for anxiolytic-like effects, we administered diazepam (1 mg/kg). Before use, diazepam (ampoule 1 mg/ml) was appropriately diluted with distilled water. Saline and Tween 80 were utilized to suspend the methanolic extract of *C. elatior*. For the Open Field Test and Hole Cross Test, animals received the methanolic extract of *C. elatior* orally (p.o.) at doses of 100, 200, and 400 mg/kg, with extract doses of 200 and 400 mg/kg applied through the same route for the Elevated Plus Maze Test and Light Dark Box Test. All experimental mice in the "Normal Control" group were administered 0.9 percent physiological saline (0.1 ml/mouse) through the same routes. Gavages were employed to administer drugs and samples to all groups.

#### 2.5.1 Elevated plus maze test

The Elevated Plus Maze (EPM) test was conducted in accordance with [29] to assess the anxiolytic effects of the sample extracts, with some modifications. The plus maze consists of four arms: two open arms (25 × 5 × 0.5 cm) and two closed arms (25 × 5 × 16 cm) with a central square creating a cross (5 × 5 × 0.5 cm) that connects the four arms. The entire maze was elevated 50 cm above the ground. Animals were individually placed at the intersection of the EPM's four arms, with one of the open arms facing them, and their behavior was observed for 5 min. The extract sample was administered 30 min prior to the experiment. The time spent in each arm and the number of entries into open arms were recorded. An entry was considered when all four paws were within either an open or closed arm. For each animal, the percentage of open-arm entries (calculated as 100 × open arm entries/total number of entries into each arm) and the total number of entries into each maze arm were determined.

#### 2.5.2 The light/dark box test

The light/dark test is based on rodents' innate sensitivity to illuminated environments and their exploratory behavior in response to moderate stress, such as exposure to new and unfamiliar surroundings. The light-dark box (LDB) model consists of a rectangular shape with walls measuring 46 × 27 × 30 cm. It features an entrance at the top that is divided into a small compartment (18 × 27 cm) and a larger section (27 × 27 cm) at ground level, separated by a transparent door (7.5 × 7.5 cm) in the middle of the divider (1/3). The small compartment is painted black on the inside and covered, while the larger compartment (2/3) is painted white. A 60 W electric lamp is suspended from the ceiling at the center of the large chamber to provide illumination. The time spent in the box during a 5-min period was monitored for each animal, with the extracted sample administered 30 min prior to the experiment [30]. When exposed to a new environment or novel stimuli, animals enter a state of natural conflict. An improvement in behavior within the white compartment, induced by the drug, is considered a measure of anxiolytic activity in the light/dark test. An increase in transitions without a corresponding rise in spontaneous locomotion is also regarded as indicative of anxiolytic action.

#### 2.5.3 Open field test

The open field test is a widely used and straightforward method for studying rodent anxiety and exploratory behavior. This method is based on anxiety models in mice and is used to record locomotor activity in a novel environment, allowing the investigation of basic and pharmacologically altered aspects of anxiety-related behavior in animals with minor modifications. The open field (OF) test was conducted in a square arena measuring 60 cm × 60 cm. The arena had a floor marked in white and green and was divided into 36 squares (10 cm × 10 cm). Continuous, 25-cm-high walls surrounded the arena, and the animals' activity was recorded. This experiment was conducted at room temperature under natural light conditions [31]. After oral administration of drug doses, animals were transferred from their home cages to the open field area. The number of squares crossed by the animals was recorded for 3 min, beginning at 30, 60, 90, and 120 min following the administration of the test medications.

#### 2.5.4 Hole cross test

To assess CNS activity with slight modifications, we conducted the hole-cross test. The hole-cross module was constructed from wood measuring (30 × 20 × 14) cm^3^ with a height of 7.5 cm. In the center of the box, there was a hole measuring 3 cm [32]. Mice were promptly placed in the box at 30-, 60-, 90-, and 120-min intervals after each group's treatment and observed for 5 min, with the number of holes crossed being recorded.

### 2.6 In silico analysis

#### 2.6.1 Ligand preparation

A total of 40 natural compounds of *Chloranthus elatior* leaves were identified [33]. The 3D structures were retrieved, except for ligand 10. All compounds’ energies were optimized and converted into the PDBQT format [34,35].

#### 2.6.2 Protein preparation

The crystal structures of the potassium channel (PDB ID: 4UUJ) [36] (Resolution: 2.40 Å, Total Structure Weight: 60.87 kDa and Modeled Residue Count: 542) were retrieved from the RCSB Protein Data Bank (http://www.rcsb.org) [37] using the protein preparation wizard in PyMOL version 1.1.0 [38]. This prepared the modeled protein for docking analysis by removing the water molecules.

#### 2.6.3 Molecular docking analysis

For the docking of the chosen protein-ligand complexes, Auto Dock Vina in PyRx version 0.8 [39] was used. The grid center points were set at X = 37.82, Y = −20.94, Z = −6.50 and dimensions (Å) to X = 40.38, Y = 57.98, Z = 59.61. In the molecular docking process, the protein was considered the macromolecule, and the ligand was considered as a ligand. After ligand docking, this ligand was optimized for maximum energy. The Discovery Studio visualization was used to assess hydrogen and hydrophobic interactions in the ligand-protein docking complex [40].

#### 2.6.4 Pharmacokinetics properties analysis

SwissADME was used to predict the pharmacokinetics of phytocompounds from ‘*Chloranthus elatior’*. This analysis focused on aspects such as absorption, distribution, metabolism, excretion, solubility, toxicity, carcinogenicity, and bioavailability, and drug-likeliness properties [41,42].

### 2.7 Statistical analysis

The data is presented as mean ± SEM. Statistical analysis was conducted using one-way analysis of variance (ANOVA), followed by Dunnett's post hoc test. In each instance, significance was set at P < 0.05. SPSS software was used for all statistical analyses.

## 3 Results

### 3.1 Phytochemical screening

Different tests were carried out to perform a preliminary phytochemical screening of MECE. Most of the tests yielded positive results, indicating the presence of alkaloids, flavonoids, tannins, saponins, cardiac glycosides, carbohydrates, and proteins in the extract. The presence and absence of secondary metabolites are presented in Table 1.

**Table 1:** Screening of the methanolic leaf extract of *Chloranthus elatior* (MECE) for phytochemicals using various assays.

Phytochemical Compounds	Name of Tests	Inference
Alkaloid	Wagner's Test	+ve
	Dragendorf's Test	
	Mayer's Test	
Flavonoids	Shinods Test	+ve
	Sodium Hydroxide Test	
	Pew's Test	
Tannins	Ferric Chloride Test	+ve
	Lead sub-acetate Test	
Phlobatannins	Precipitate Test	-ve
Saponins	Foam Test	+ve
Steroids	Salkowski Reaction	-ve
Cardiac Glycoside	Keller Kelliani's Test	+ve
Carbohydrate	Molisch's Test	+ve
Protein	Biuret Test	-ve

### 3.2 Elevated plus maze test (EPM)

The outcomes of this test are presented in Table 2. The study showed that the number of entries (1.5 ± 0.75 at a dose of 400 mg/kg and 1.29 ± 0.65 at a dose of 200 mg/kg) in the open arm were remarkably greater in comparison with those of results seen in the closed arm at both doses. Similar results (for time spent) were obtained in the case of the open arm as well.

**Table 2:** The effect of MECE and standard drug on anxiety during the elevated plus maze (EPM) test.

Obj. no.	Treatments	No. of entries in closed arm	No. of entries in open arm	Time (Sec) spent in close arm	Time (Sec) spent in open arm
1	Standard 1 mg/ml	0.95 ± 0.48	2.38 ± 1.19	3.09 ± 1.55	3.36 ± 1.68
2	Control	2.16 ± 1.08	1.29 ± 0.65	14.76 ± 7.38	23.60 ± 11.80
3	200 mg/kg	0.96 ± 0.48∗∗	1.29 ± 0.65	4.546 ± 2.27	11.24 ± 5.62
4	400 mg/kg	0.82 ± 0.40∗∗	1.5 ± 0.75	2.58 ± 1.29	3.29 ± 1.65

All values are expressed as mean ± SEM (n = 5); One way Analysis of Variance (ANOVA). ∗∗P < 0.001, significant compared to control.

### 3.3 Light dark box test

The outcomes of this test demonstrate notable anxiolytic effects of MECE assessed using light and dark boxes. The study revealed that there was a significant decrease in the time spent in the dark chamber at both doses. However, the treatment using 200 mg/kg body weight fared optimally between the two doses tested and was better than that exhibited by the standard drug diazepam (Table 3).

**Table 3:** The effect of MECE and standard drug on anxiety during the light dark box test.

Obj. no.	Treatments	Spent time in the dark chamber
1	Control	21.79 ± 10.89
2	Standard 1 mg/ml	8.73 ± 4.37
3	Extract 200 mg/kg	5.97 ± 2.98∗∗
4	Extract 400 mg/kg	7.33 ± 3.66∗∗

All values are expressed as mean ± SEM (n = 4); significance at ∗∗P < 0.001 as compared to control.

Dunnett test using ONE-WAY-ANNOVA is used for the statistical analysis.

### 3.4 Open field test

After the administration of MECE at 100, 200, and 400 mg/kg body weight, there was a slight reduction in the number of crossing and rearing by the mice. Specifically, a suppressive effect was observed at the 400 mg/kg dose after 30 min of oral dosing (Table 4).

**Table 4:** The sedative effect of MECE and standard drug during the open field test.

Open Field Test				
Treatment	30min	60min	90min	120min
Normal control	57.85 ± 28.93	39 ± 19.5	40.05 ± 20.02	41.24 ± 20.62
Standard 1 mg/ml	23.27 ± 11.63	31.19 ± 15.59	23.96 ± 11.98	24.97 ± 12.48
Extract 100 mg/kg	28.27 ± 14.14	77.77 ± 38.88	41.45 ± 20.73	28.76 ± 14.38
Extract 200 mg/kg	17.86 ± 8.93	19.62 ± 9.81	42.98 ± 21.49	25.15 ± 12.58
Extract 400 mg/kg	6.928 ± 3.46∗	35.42 ± 17.71	31.49 ± 15.75	22.65 ± 11.32

All values are expressed as mean ± SEM (n = 5); One way Analysis of Variance (ANOVA). ∗P < 0.05, significant compared to control.

### 3.5 Hole cross test

Regarding the hole cross test, 100,200 and 400 mg/kg doses were administered. The number of hole crosses significantly decreased after 30 min of MECE treatment at 200 mg/kg. A significant alteration in movements was noted at 60 min after the oral administration of 400 mg/kg dose (P < 0.05) (Table 5).

**Table 5:** The sedative effect of MECE and standard drug during the hole cross test.

Hole Cross Method				
Treatment	30min	60min	90min	120min
Normal control	5.45 ± 2.72	3.56 ± 1.78	3.5 ± 1.75	6.65 ± 3.33
Standard 1 mg/ml	1.71 ± 0.85	0	0	1.11 ± 0.55
Extract 100 mg/kg	1.55 ± 0.77	2.29 ± 1.14	2.35 ± 1.17	2.59 ± 1.299
Extract 200 mg/kg	4.08 ± 2.04	2.5 ± 1.25	3.69 ± 1.85	2.22 ± 1.11
Extract 400 mg/kg	1.71 ± 0.85∗	0	0	0

All values are expressed as mean ± SEM (n = 5); One way Analysis of Variance (ANOVA). ∗P < 0.05, significant compared to control.

### 3.6 Molecular docking analysis for anxiolytic study

The results of the docking analysis for anxiolytic activity are presented in Table 6. Molecular docking analysis was performed on a total of 40 natural compounds of *C. elatior* leaves, docked against the 4UUJ protein. The highest docking score obtained was −8.8 kcal/mol (L-10), while the lowest score was −3.8 kcal/mol (L-24) (Table S1). Interestingly, 10 out of 40 compounds exhibited docking scores ranging from −8.8 to −6.1 kcal/mol. These ten highly potential anti-anxiolytics were further studied to assess and confirm their viability as potential lead compounds for anxiolytic research.

**Table 6:** The binding energy (in kcal/mol) of the top ten compounds with the potassium channel protein.

Ligand No	Name of Compound	PubChem CID	Docking Score (kcal/mol)
L-10	Chloramultilide C	24763324	−8.8
L-17	4-dimethoxyflavanone	15227613	−6.5
L-1	Neolitacumone B	11776782	−6.4
L-3	Chlorantholide A	101574495	−6.3
L-6	Chlorantholide D	101574498	−6.3
L-7	Chlorantholide F	101574500	−6.3
L-22	Chloranthalactone B	15767607	−6.3
L-4	Chlorantholide B	101574496	−6.2
L-15	Flavokawain A	5355469	−6.2
L-9	Zedoalactone A	15226639	−6.1

### 3.7 Protein-ligand interplay

The interaction between the active sites of ligands and proteins was evaluated following molecular docking. According to Table 7, two types of connections linked the proteins and ligands: hydrogen bonding connections and hydrophobic interactions. The primary reason for the strong hydrogen bond effect is the shorter bond distance, which measures 2.5 Å, in contrast to other bond distances that are greater than 3.1 Å. Conversely, certain types of hydrophobic bonds are considered weak bonds, as their bond distances fall within the range of 3.1 Å to 4.5 Å [43]. In this study, it was observed that the number of hydrophobic interactions was two to three times greater than the number of hydrogen bonds for each protein-ligand interaction, resulting in a higher binding affinity value.

**Table 7:** The docking interactions of the top ten compounds with the potassium channel protein.

SL no.	Compound	Hydrogen connection		Hydrophobic connection	
		Residues	Distance (A°)	Residues	Distance (A°)
1.	L-10	THR-113	2.58	GLY-42	2.29
		GLU-153	2.90	PRO-41	4.70
		VAL-157	2.75	ALA-173	5.15
2.	L-17	VAL-93	3.03	PRO-41	4.79
		TYR-180	2.40	GLU-153	3.36
3.	L-1	GLU-62 GLU-50	2.90 2.00	TRP-47	4.54
				LYS-63	3.79
				HIS-35	5.39
4.	L-3	TYR-180	2.72	PRO-41	4.75
				VAL-93	4.62
5.	L-6	HIS-35	3.62	TRP-47	4.35
6.	L-7	PRO-131	3.40	LEU-129	4.61
7.	L-22	TYR-180	2.59	PRO-41	4.57
		GLU-153	3.28	VAL-93	4.66
8.	L-4	Absent	Absent	TRP-47	4.35
9.	L-15	SER-91	2.08	PRO-41	4.05
		SER-117	2.69		
		TYR-180	3.42		
10.	L-9	SER-91	3.61	PRO-41	4.57
				GLU-153	2.88

**Fig. 1:**
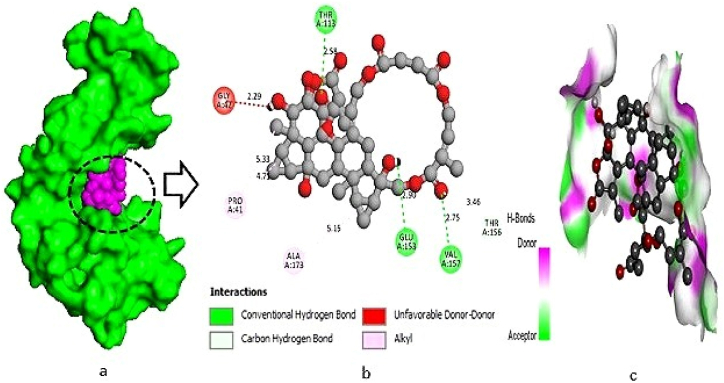
Docking between the protein‐ligand interplay of potassium channel protein (PDB ID: 4UUJ) and Chloramultilide C (L-10), where (a) Surface sight by PyMOL (binding pocket in active site), (b) 2D observation using discovery studio, (c) Hydrogen bonding.

**Fig. 2:**
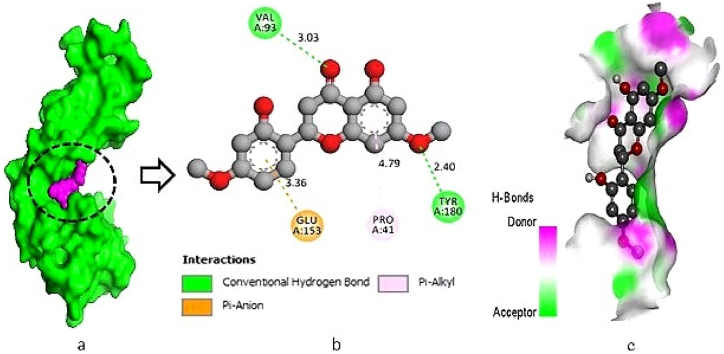
Docking between the protein‐ligand interplay of potassium channel protein (PDB ID: 4UUJ) and 4-dimethoxyflavanone (L-17), where (a) Surface view by PyMOL (binding pocket in active site), (b) 2D observation using discovery studio, (c) Hydrogen bonding.

**Fig. 3:**
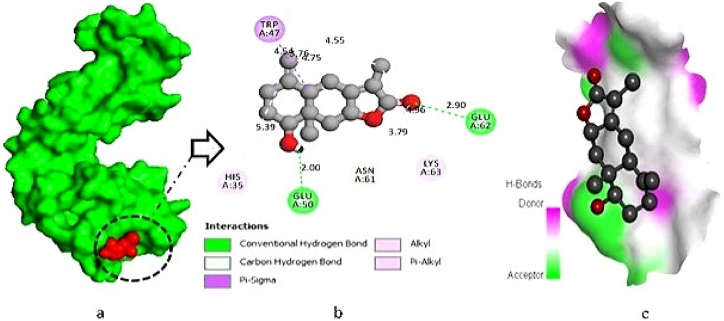
Docking between the protein‐ligand interplay of potassium channel protein (PDB ID: 4UUJ) and Neolitacumone B (L-01), where (a) Surface view using PyMOL (binding pocket in active site), (b) 2D observation using discovery studio, (c) Hydrogen bonding.

The L-10 compound (Chloramultilide C) exhibited a total of six protein-ligand interactions, including three conventional hydrogen bonds with the following positions: THR-113 (2.58 Å), GLU-153 (2.90 Å), and VAL-157 (2.75 Å). Additionally, three hydrophobic bonds were observed at GLY-42 (2.29 Å), PRO-41 (4.70 Å), and ALA-173 (5.15 Å) (Fig. 1a–c). For the L-17 compound (4-dimethoxyflavanone), two conventional hydrogen bonds were identified at the binding residues VAL-93 (3.03 Å) and TYR-180 (2.40 Å), along with two hydrophobic bonds formed with the potassium channel protein at binding residues PRO-41 (4.79 Å) and GLU-153 (3.36 Å) (Fig. 2a–c). In the interaction study of L-01 (Neolitacumone B), ten conventional hydrogen bonds were observed at the positions of GLU-62 (2.90 Å), GLU-50 (2.00 Å), and TRP-47 (4.54 Å), as well as LYS-63 (3.79 Å) and HIS-35 (5.39 Å) forming hydrophobic bonds (Fig. 3a–c).

### 3.8 Molecular properties

Table 8 presents the molecular weight, number of H-bond acceptors, number of H-bond donors, log S, topological polar surface area (TPSA), molar refractivity, bioavailability score, and human intestinal absorption data for the top ten lead compounds resulting from the investigation of molecular properties. The results indicated that all compounds had molecular weights of less than 500 Da, with the exception of L-10. It is worth noting that to qualify as a drug candidate, the topological polar surface area (TPSA) score must typically fall within the range of 0–140 [36]. In the case of these compounds, the TPSA values ranged from 38.83 to 85.22, except for L-1. The bioavailability score was consistently high, approximately around 0.55, and the molar refractivity values ranged from 64.85 to 172.71.

**Table 8:** Molecular properties of the top ten selected compounds from *Chloranthus elatior* leaf extract.

Ligand no.	Molecular Weight	H-bond acceptors	H-bond donors	Fraction Csp3 (0.25)	TPSA(Å^2^) (20–130)	Log S (ESOL) mol/L	Molar Refractivity	Bioavailability Score
L-10	734.70	14	4	0.66	212.42	−3.82	172.71	0.17
L-17	316.30	6	2	82.53	85.22	−3.44	82.53	0.55
L-01	248.30	3	1	0.67	46.53	−3.07	69.12	0.55
L-03	244.29	3	0	0.47	43.37	−3.15	67.68	0.55
L-06	262.30	4	1	0.60	63.60	−2.90	69.35	0.55
L-07	278.30	5	2	0.60	83.83	−3.12	70.55	0.55
L-22	244.29	3	0	0.60	38.83	−2.24	64.85	0.55
L-04	246.30	3	0	0.60	43.37	−3.11	68.15	0.55
L-15	314.33	5	1	0.17	64.99	−4.92	87.75	0.55
L-09	266.33	4	2	0.80	66.76	−2.72	71.09	0.55

**Note:** Molecular Weight (acceptance range: <500), HBD = hydrogen bond donor (acceptance range: ≤5), HBA = hydrogen-bond acceptor: (acceptance range: ≤10).

### 3.9 Pharmacokinetics studies

Pharmacokinetics, also known as absorption, distribution, metabolism, excretion, and toxicity (ADMET), is the study of how pharmaceuticals enter, traverse, and exit the body. The lipophilicity of these compounds falls within the range of 0.99–2.82. For nine out of the ten drugs, human intestinal absorption exceeds 92 %. Six of the compounds exhibit positive blood-brain barrier (BBB) permeability, while all ten compounds show negative central nervous system (CNS) permeability. There are no observed hepatotoxic effects (Table 9).

**Table 9:** ADMET prediction of the top ten selected compounds from *Chloranthus elatior* leaf extract.

L/N	Absorption		Distribution		Metabolism		Excretion		Toxicity	
Ligand no.	Lipophilicity	Intestinal absorption (human) Yes/No 75 %+	BBB permeability Yes/No	CNS Permeability (log PS)	CYP3A4substrate (Yes/No)	CYP3A4 inhibitor (Yes/No)	Total Clearance(ml/min/kg)	Renal OCT2 substrate((Yes/No)	Max. Tolerated dose (human) mg/kg/day	Hepatotoxicity
L-10	0.99	98.59	−1.10	−3.22	Yes	No	−0.77	No	−1.63	No
L-17	2.82	92.29	−0.39	−2.94	No	Yes	0.52	No	0.55	No
L-01	2.30	95.80	0.36	−2.86	No	No	1.12	No	0.35	No
L-03	2.68	99.06	0.09	−2.73	No	No	0.58	No	0.18	No
L-06	1.88	97.21	0.07	−2.88	No	No	0.96	No	0.27	No
L-07	0.99	75.19	0.11	−2.98	No	No	1.00	No	0.40	No
L-22	2.18	99.07	0.65	−2.43	Yes	No	0.47	Yes	0.18	No
L-04	2.56	99.38	0.09	−2.73	No	No	1.05	No	0.19	No
L-15	3.31	92.32	−0.32	−2.36	Yes	Yes	0.32	No	0.89	No
L-09	1.55	95.96	−0.24	−2.97	No	No	1.01	No	0.44	No

Note: Log P = High Lipophilicity (acceptance range: <5).

## 4 Discussion

This study delved into the effects of a methanolic extract of *C. elatior* on the central nervous system (CNS) of mice, representing the first comprehensive investigation into the psychoactive potential of this *Chloranthus* species. Neuropharmacological test methods, such as the elevated plus maze, open field, and hole cross test, as well as light-dark box tests, were employed to scrutinize the influence of *C. elatior* on the central nervous system.

Firstly, we examined the impact of MECE and diazepam on elevated plus maze activities. The elevated plus maze test is a well-established paradigm for assessing anxiety in animals, capitalizing on their innate conflict between the fear of open spaces and the drive to explore new environments. Grooming behavior is a reliable indicator in this test for prognostic validity. Following oral administration of *C. elatior* extract (400 mg/kg), we observed a gradual increase (p < 0.001) in the time spent in the open arms of the EPM, indicating an anxiolytic effect. The diagonal crossing was monitored to account for general changes in locomotion, and no significant alterations were detected following MECE or diazepam treatment. According to Mishra et al. (2011) the EPM test result is also similar to this experiments result. Hence, this suggests that MECE exhibits sedative properties [44].

The light-dark box (LDB) test is based on rodents' natural tendency to seek well-lit areas and engage in exploratory behaviors when confronted with mild disturbances, such as light and novel surroundings. Some reports suggest that the most reliable criterion for assessing anxiolytic activity is not the number of transitions but rather the time spent in the light area [45,46]. The anxiolytic effect of MECE became evident as we observed that MECE treatment prompted animals to spend more time in the open and well-lit sections of both the EPM and LDB.

To assess the impact of the extract on spontaneous locomotor activity, we conducted the open field test (OFT). Our results demonstrated that MECE significantly reduced spontaneous locomotor activity in mice compared to control groups at a dose of 400 mg/kg (P < 0.05). This reduction suggests that MECE possesses sedative characteristics. The hole cross test was another method employed to evaluate anxiety-like behavior, and a marked decrease in the number of hole crosses was observed at the 400 mg/kg dose. This suggests that *C. elatior* may exert an anxiolytic effect. The consistent outcomes of the EPM, LDB, and OFT tests support the conclusion that MECE exhibits a robust anxiolytic effect. The probable mechanism for this anxiolytic effect may resemble that of commercial drugs. MECE is likely to potentiate chloride ion transport mediated by GABA, thereby enhancing its inhibitory effects [47]. Moreover, the reported anxiolytic action of *C. elatior* could be attributed to an agonistic effect on the GABA/benzodiazepine receptor complex, antagonizing the 5-HT1B receptor, or agonizing the 5-HT1A receptor [48]. Qualitative phytochemical analysis of MECE revealed the presence of alkaloids, glycosides, carbohydrates, flavonoids, and tannins. Other plant extracts containing alkaloids, flavonoids, tannins, and terpenoids have been known to possess potent sedative and anxiolytic effects in Swiss albino mice [23]. Although the precise contributions of these various components to the sedative and anxiolytic effects of MECE have not been fully elucidated, it is plausible that these purportedly neuroactive substances may, to some extent, underlie the mechanism behind these effects.

The potential anxiolytic activities of MECE warrant further investigation into the underlying mechanisms for the development of new drugs. Molecular docking studies were conducted to explore the binding modes of these compounds with target receptors. Several bioactive compounds have been identified within the Chloranthceae family, and *C. elatior*, an important species within this family with potential anxiolytic properties, exhibited significant interactions with a target protein, the potassium channel (PDB ID: 4UUJ). In a previous investigation, compounds identified from the methanolic extract of *Piper nigrum* exhibited differentiated docking scores ranging from −1.0 to −7.90 kcal/mol, where Piperolactam D showed the highest binding affinity (−7.9 kcal/mol) with the potassium channel (PDB ID: 4UUJ) [36]. In another previous study, the lead compound 3,4-Dihydroxybenzoic acid from the *Vitex peduncularis* Wall. leaves demonstrated anxiolytic activity by binding with the potassium channel receptor with a −4.81 kcal/mol binding affinity [49]. Besides, the phytoconstituent 4H-pyran-4-one, 2,3-dihydro-3,5-dihydroxy-6-methyl- from the leaf extract of *Cnesmone javanica* Blume presented the highest binding affinity (−4.887 kcal/mol) with the potassium channel [50]. Compounds from *Vitex peduncularis* also showed anxiolytic activity, where Vitexin demonstrated −4.305 kcal/mol binding affinity as the top molecule binding with the potassium channel receptor [51]. Phytyl acetate from *Tetrastigma leucostaphyllum* was the top molecule to show anxiolytic activity which disclosed a −5.8 kcal/mol docking score with the potassium channel receptor [52]. Moreover, Vanillin from *Duabanga grandiflora* (DC.) Walp. stem bark and Psychotriasine from the methanolic extract of *Psychotria calocarpa* leaves exhibited −4.60 kcal/mol and −3.359 kcal/mol docking score as the top molecule binding with the potassium channel receptor, respectively [53,54]. In this investigation, we found that 10 compounds displayed substantial binding affinity with the target protein among the 40 compounds from the Chloranthceae family, with docking scores ranging from −8.8 to −6.1 kcal/mol. This suggests that these top 10 phytochemicals are responsible for the anxiolytic activity by binding to the 4UUJ protein and also showed better results than these previous studies. Furthermore, we employed the SwissADME online tool to assess the toxicological parameters of the assigned plant and explore its safety [55]. The results indicate that these compounds are safe and adhere to Lipinski's rules, suggesting their potential as drug candidates.

## 5 Conclusion

Throughout history, medicinal plants have consistently served as a dependable source of affordable and effective remedies. Many ethnomedicinal plants have been observed to influence neurobehavioral states and complement conventional medicine. Our current research unequivocally demonstrates that the phytoconstituents of *C. elatior* indirectly suggest potential in the treatment of anxiety disorders. Further in-depth studies are required to identify the bioactive phytochemical(s) and comprehend the specific molecular mechanisms underlying the observed pharmacological activities. A computational study validates the experimental results and increases interest in the identification of new compounds for the development of novel drugs. Additionally, we believe that this research will draw more attention to the effects of the methanolic leaf extract of *C. elatior* on the central nervous system and provide insight and knowledge for the treatment of anxiety in the approach to medicinal plants and a foundation for future investigations into MECE (Methanolic Extract of *C. elatior*).

## CRediT authorship contribution statement

**Umme Tabassum Arobi Katha:** Writing – original draft, Formal analysis, Data curation, Conceptualization. **Yesmin Begum:** Writing – review & editing, Supervision, Software, Project administration, Conceptualization. **Md Golam Mortuza:** Methodology, Formal analysis, Data curation. **Sayma Sharmin:** Methodology, Investigation, Data curation. **Md Rafiquzzaman:** Methodology, Formal analysis, Data curation. **Suvro Biswas:** Methodology, Investigation, Formal analysis. **Md Abu Saleh:** Writing – review & editing, Supervision, Project administration, Conceptualization.

## Ethical approval

The study procedure was accepted (SEU/Pharm/CECR/113/2023, Dated: 10.07.2023) by the Ethics Committee of the Department of Pharmacy, Southeast University, Dhaka, Bangladesh. The study was conducted according to the ethical standards established in the 1964 Declaration of Helsinki.

## Data and code availability statement

Data included in article/supplementary material is referenced in the article.

## Funding source

This work received no external funding.

## Declaration of competing interest

The authors declare that they have no known competing financial interests or personal relationships that could have appeared to influence the work reported in this paper.
