# Supplementary material for: Phytoconstituents of Chloranthus elatior as a potential adjunct in the treatment of anxiety disorders: In vivo and in silico approaches
Source: Heliyon. 2024 Nov 28;10(23):e40728. doi: 10.1016/j.heliyon.2024.e40728 (PMC11652921; doi:10.1016/j.heliyon.2024.e40728)
Supplement: Multimedia component 1 [file mmc1.docx]

**Phytoconstituents of *Chloranthus elatior as a potential adjunct in the treatment of anxiety disorders: in vivo and in silico approaches***

Umme Tabassum Arobi Katha^1^, Yesmin Begum^1,^*, Md. Golam Mortuza^2^_,_ Sayma Sharmin^1^, Md. Rafiquzzaman^3^, Deri Damien Hagidok^4^, Suvro Biswas^4^, Md. Abu Saleh^4,*^

^1^Department of Pharmacy, Southeast University, Dhaka, Bangladesh.

^2^Department of Science and Humanities, Bangladesh Army International University of Science and Technology, Cumilla 3500, Bangladesh.

^3^Department of Pharmacy, Jahangirnagar University, Savar, Dhaka, Bangladesh.

^4^Microbiology Laboratory, Department of Genetic Engineering and Biotechnology, University of Rajshahi, Rajshahi-6205, Bangladesh.

*Corresponding author: Yesmin Begum: [yeasmin.begum@seu.edu.bd](mailto:yeasmin.begum@seu.edu.bd)

Md. Abu Saleh: [saleh@ru.ac.bd](mailto:saleh@ru.ac.bd)

**Table S1:** Molecular docking score between ***Chloranthus elatior*** *leaf extract* compounds and selected protein.

| **Ligand No** | Name Of Molecule | CID | Docking Score **(kcal/mol)** |
| --- | --- | --- | --- |
|  | Neolitacumone B | 11776782 | -6.4 |
|  | Chloranthalic Acid | 101611296 | -6.0 |
|  | Chlorantholide A | 101574495 | -6.3 |
|  | Chlorantholide B | 101574496 | -6.2 |
|  | Chlorantholide C | 101574497 | -6.0 |
|  | Chlorantholide D | 101574498 | -6.3 |
|  | Chlorantholide F | 101574500 | -6.3 |
|  | Shizukolidol | 91884869 | -6.3 |
|  | Zedoalactone A | 15226639 | -6.1 |
|  | Chloramultilide C (Henriol A) | 405431835 | -8.8 |
|  | Eudesmane | 193299 | -5.6 |
|  | Guaiane, | 9548703 | -5.6 |
|  | Germacrane, | 9548707 | -5.7 |
|  | Cadinane, | 9548708 | -5.3 |
|  | Flavokawain A; | 5355469 | -6.2 |
|  | Ferulic Aldehyde | 5280536 | -5.2 |
|  | 4-Dimethoxyflavanone | 15227613 | -6.5 |
|  | Diastereoisomers Of Dinor-Eudesmenes, | 71751946 | -6.0 |
|  | P-Coumaric Acid Or ß-Coumaric Acid | 637542 | -5.0 |
|  | Trihydroxyaromadendrane | 15761787 | -5.2 |
|  | 4- Dihydroxymaaliane | 10823866 | -5.8 |
|  | Chloranthalactone B | 15767607 | -6.3 |
|  | Sabinene | 18818 | -4.6 |
|  | Myrcene | 31253 | -3.8 |
|  | Linalool | 6549 | -4.0 |
|  | Neo-Alloocimene | 5371125 | -4.5 |
|  | Geraniol | 637566 | -4.3 |
|  | Alloaromadendrene | 91354 | -5.8 |
|  | Gamma-Muurolene | 12313020 | -5.4 |
|  | Germacrene D | 5317570 | -5.5 |
|  | Amorpha-4 | 11052747 | -5.5 |
|  | Delta-Amorphene | 12306059 | -5.7 |
|  | Germacrene B | 5281519 | -5.7 |
|  | Germacrone; | 6436348 | -5.6 |
|  | Α –Terpinene | 198978036 | -4.8 |
|  | Α -Copaene; | 198963142 | -5.7 |
|  | Β –Elemene | 250138823 | -4.9 |
|  | Β –Longipinene | 249993285 | -5.6 |
|  | Α –Gurjunene | 348291514 | -5.8 |
|  | Α –Humulene; | 249979180 | -5.2 |
